# Zebrafish study provides evidence for *Porphyromonas gingivalis* outer membrane vesicles eliciting Alzheimer’s disease-like pathologies

**DOI:** 10.3389/fcimb.2026.1761068

**Published:** 2026-03-06

**Authors:** Jianbin Guo, Kaijin Lin, Ruiyun Liang, Lingxia Jiang, Xiaozhen He, Jiawei Chen, Minqian Zheng

**Affiliations:** 1 Clinical Research Center for Oral Tissue Deficiency Diseases of Fujian Province & Fujian Key Laboratory of Oral Diseases & Fujian Provincial Engineering Research Center of Oral Biomaterial, School and Hospital of Stomatology, Fujian Medical University, Fuzhou, China; 2 Stomatological Key laboratory of Fujian College and University & Institute of Stomatology & Research Center of Dental and Craniofacial Implants, School and Hospital of Stomatology, Fujian Medical University, Fuzhou, China; 3 Institute of Life Sciences, College of Biological Science and Engineering, Fuzhou University, Fuzhou, China

**Keywords:** periodontitis, alzheimer’s disease, porphyromonas gingivalis, outer membrane vesicle, Zebrafish

## Abstract

**Introduction:**

Periodontitis has been epidemiologically linked to an increased risk of Alzheimer’s disease (AD), yet the mechanistic contribution of periodontal pathogens remains insufficiently understood. Building on our previous findings that *Porphyromonas gingivalis* outer membrane vesicles (OMVs) induce cardiovascular dysfunction, this study investigates whether these vesicles also drive AD‐related pathology using the zebrafish model.

**Methods:**

We microinjected *P. gingivalis* OMVs into the common cardinal vein of zebrafish larvae to evaluate locomotor behavior, brain injury, and neuroinflammatory responses. Integrated proteomic and transcriptomic analyses were performed to identify alterations in AD‐associated pathways, and acetylcholinesterase activity along with Aβ1–42 plaque accumulation were quantified to validate hallmark AD phenotypes.

**Results:**

OMV exposure resulted in significant neurotoxicity, locomotor deficits, and robust neuroinflammation, accompanied by pronounced dysregulation of AD-related molecular pathways. Notably, OMVs markedly increased acetylcholinesterase activity and promoted Aβ1–42 deposition in larval brains.

**Discussion:**

These findings demonstrate that *P. gingivalis* OMVs act as potent inducers of neuronal damage and AD-like pathological features *in vivo*, providing mechanistic insight into how periodontal pathogens may contribute to neurodegenerative disease progression.

## 1 Introduction

Periodontitis, a prevalent chronic inflammatory disease affecting the supporting structures of the teeth, has been increasingly implicated in the pathogenesis of Alzheimer’s disease (AD) and related neurodegenerative disorders. Multiple epidemiological studies have demonstrated a positive association between long-standing periodontitis and an elevated risk of developing AD (Liu et al., 2018). Importantly, this association is supported by *in vivo* evidence. Chronic oral application of Porphyromonas gingivalis in wild-type mice has been shown to induce brain inflammation, neurodegeneration, and extracellular Aβ1–42 deposition, together with tau hyperphosphorylation, recapitulating key pathological features of Alzheimer’s disease (Ilievski et al., 2018). Notably, a retrospective cohort analysis revealed that individuals with chronic periodontitis persisting for more than a decade exhibited a significantly higher incidence of AD compared to periodontally healthy individuals, underscoring the potential systemic consequences of oral inflammation (Villar et al., 2024). The biological plausibility of this association is supported by two primary mechanistic pathways. First, direct bacterial invasion of the central nervous system has been demonstrated for keystone periodontal pathogens such as *Porphyromonas gingivalis* (*P. gingivalis*), which can translocate to the brain and release virulence factors—including lipopolysaccharide (LPS) and gingipains—that promote β-amyloid (Aβ) aggregation, tau hyperphosphorylation, and cholinergic dysfunction (Dominy et al., 2019). Second, systemic inflammation induced by chronic periodontitis may disrupt the integrity of the blood–brain barrier (BBB), activate microglia, and exacerbate neuroinflammatory cascades, thereby accelerating neuronal loss and cognitive decline (Costa et al., 2021).

While substantial evidence has focused on whole bacteria and soluble virulence factors, comparatively little is known about the role of *P. gingivalis* outer membrane vesicles (OMVs) in neurodegenerative processes. OMVs, nanoscale extracellular structures enriched in proteins, lipids, and enzymes, are capable of penetrating host barriers and delivering concentrated virulence payloads to distant tissues (Walker et al., 2023). Our previous work demonstrated that *P. gingivalis* OMVs induce pronounced cardiovascular damage in zebrafish, characterized by endothelial dysfunction, pericardial edema, and aberrant angiogenesis (Guo et al., 2024). Given the established contribution of *P. gingivalis* to neuroinflammation, it is plausible that OMVs may similarly exert neurotoxic effects through direct interaction with neural tissues or systemic inflammatory pathways.

In this study, a zebrafish model was employed to investigate the potential link between *P. gingivalis* OMVs and AD. Zebrafish were exposed to *P. gingivalis* OMVs, followed by comprehensive analyses including neurophenotypic characterization, behavioral assessment, and proteomic profiling. These findings are expected to provide experimental evidence supporting the association between periodontitis and increased AD risk, offering valuable insights for the development of preventive and therapeutic strategies for related neurodegenerative disorders.

## 2 Materials and methods

### 2.1 Preparation, identification and observation of *P. gingivalis* OMVs

For OMV isolation, freshly cultivated bacteria at an optical density of 600 (OD 600 = 1), which corresponded to 9 × 10^9^ colony-forming units (CFU), were subjected to centrifugation at 8,000 g and 4°C for 5 min, resulting in the collection of particles. Filter the upper liquid (0.2 μm) and further centrifuge 1 h at 100,000 g at 4°C. After isolation, OMV particles were washed, suspended in PBS, and subjected to analysis and characterization using a transmission electron microscope (TEM, Hitachi, HT-7700) and a nanoparticle size analyzer (NSA, Nano FCM, N30E). The protein concentration of OMVs was determined using the BCA protein concentration kit (TIANGEN, PA115). The yield of OMVs, expressed as the amount of protein (µg) obtained per 1010 bacteria, was calculated based on the bacterial count corresponding to OD 600 (5.6 × 10^10^ CFU/mL). The average diameter of P. gingivalis OMVs was about 72.28 nm with a concentration of 8.19 × 10^10^ particles/mL.

### 2.2 Culture of *P. gingivalis*

Wild-type *P. gingivalis* strain ATCC 33277 was inoculated into brain-heart infusion broth (Oxoid) containing 5 mg/mL yeast extract, 250 μg/mL L-cysteine, 1 mg/mL hemin, and 1 mg/mL vitamin K and incubated anaerobically (37°C, 80% N2, 10% CO2, and 10% H2) (Choi et al., 2014; Nakao et al., 2014).

### 2.3 Animal handling

WT AB strain zebrafish, *Tg (elavl3:EGFP)* and *Tg (mpx:EGFP)* (from the China Zebrafish Resource Center, Wuhan, China; http://zfish.cn) were maintained and raised according to the Zebrafish Book (Westerfield 2000). *Tg (nfkb:EGFP)* (from Fuzhou bio-service Biotechnology Co., Ltd. Fuzhou, China) was constructed follow the report (Furuyama and Sircili, 2021). All zebrafish work was conducted under full animal care and use guidelines with prior approval by the local institutional animal care and use committee (Institutional Animal Care and Use Committee, Fujian Medical University 2025-Y-0165).

### 2.4 Treatment of zebrafish with *P. gingivalis* OMVs

At 48 hours post-fertilization (hpf), zebrafish embryos were dechorionated with help of a sharp tip forceps and anesthetized with 0.04 mg/ml of tricaine (MS-222, Sigma). Anesthetized embryos were transferred onto a modified agarose gel for microinjection. Approximately 4 nL of PBS or OMVs (0, 1.25, 2.5 and 4.5 μg/μL) derived from wild-type *P. gingivalis* were injected into the common cardinal vein of each embryo using an Eppendorf microinjector (FemtoJet 4i, Eppendorf) (Widziolek et al., 2016; Farrugia et al., 2020). For each concentration, n= 30 embryos per group were injected, and experiments were repeated in three independent biological replicates (total n ≥ 90 embryos per group). At 2, 24, and 48 h post-injection (hpi), live imaging was performed under a stereoscopic fluorescence microscope (SMZ800N) and evaluated with the Image J software.

### 2.5 Behavior testing

At 48 hpf, zebrafish larvae were subjected to a Touch-evoked escape response (TER) assay to evaluate locomotor behavior. A fine needle tip was used to gently touch the posterior trunk or tail of each larva. Larvae that failed to move a distance at least three times their body length upon stimulation were considered to exhibit locomotor impairment. A movement diagram was recorded for both groups to assess the motility changes caused by *P. gingivalis* OMVs. For each treatment group, at least 15 larvae were analyzed per experiment, and the assay was independently repeated three times. Larval movements were captured under identical conditions using a digital camera mounted on a stereomicroscope. The scale bar was set to 0.5 mm.

### 2.6 Trajectory analysis

The movement trajectories of zebrafish larvae were analyzed based on the photographs taken after the treatment with *P. gingivalis* OMVs or PBS. Larvae were observed for movement behavior using the Daniovision Observation System (Noldus, Netherlands). Trajectories were analyzed with EthoVision XT 17 software (Noldus), which calculated total swimming distance and average velocity. The total distance traveled and the speed of movement were recorded for each individual larva over the observation period. A minimum of 15 larvae per group per experiment were included in the analysis, with three independent biological replicates performed for each condition. The movement patterns were quantified and compared between the OMVs-treated group and the control group.

### 2.7 Combined analysis of proteomics and transcriptomics

#### 2.7.1 Transcriptomic analysis

Zebrafish larvae treated with PBS or OMVs (2.5 μg/μL) were collected and used for transcriptional profiling at 48 hpi. Each group consisted of three independent biological replicates,with approximately 50 larvae pooled per replicate. Total RNA was extracted from tissue samples. Concentration and purity were determined using the NanoDrop 2000 (OD260/280 ratio of 1.8–2.2). RNA integrity was assessed via agarose gel electrophoresis and Agilent 5300 analysis (RQN > 6.5), ensuring total RNA quantity ≥ 1 μg and concentration ≥ 30 ng/μL. Use Oligo(dT) magnetic beads to enrich mRNA containing polyA tails, then randomly fragment them into approximately 300 bp fragments using Fragmentation Buffer. Synthesize double-stranded cDNA using random primers, perform end repair and A-base addition, then ligate adapters. Screen for target fragments and perform PCR amplification to construct the library. The library is quantified using Qubit 4.0, mixed, and processed by the cBot system to generate sequencing clusters via bridge PCR. Finally, high-throughput paired-end sequencing is performed on the NovaSeq X Plus platform to obtain raw transcriptomic data.

#### 2.7.2 Proteomics analysis

Proteomics analysis was performed on zebrafish head tissue treated with the extract. For each group, head tissues from approximately 50 larvae were pooled as one biological replicate, with three independent replicates analyzed. First, proteins were extracted and quantified (Bradford/BCA method), and sample quality was assessed by SDS-PAGE. Take 100 μg of protein, add TEAB (100 mM) sequentially, followed by TCEP (10 mM, 37°C for 60 min) to reduce disulfide bonds, and IAM (40 mM, room temperature, protected from light for 40 min) to block sulfhydryl groups. Centrifuge to collect the precipitate and redissolve it in TEAB. Add trypsin at a ratio of enzyme/protein = 1:50 and incubate at 37°C overnight. Peptides were vacuum-concentrated, resuspended in 0.1% TFA, desalted using HLB, and then concentrated and quantified (NanoDrop One). Finally, mass spectrometry analysis was performed in DIA mode (Thermo Xcalibur 4.7, Astral mass spectrometer, Vanquish Neo chromatography system), combined with Spectronaut™ software analysis, to complete protein qualitative and quantitative analysis and subsequent bioinformatics statistics.

#### 2.7.3 Combined analysis of transcriptomes and proteomes

Differentially expressed proteins and mRNAs were extracted from the joint analysis of proteomes and transcriptomes, and the results were subjected to correlation clustering analysis and GO/KEGG enrichment analysis.

### 2.8 Determination of acetylcholinesterase activity

For each group, head tissues from 50 embryos were pooled as one sample. The assay was performed using three independent biological replicates per group. Their heads were washed with cold PBS (0.01M, pH=7.4) and weighed. The head tissues were homogenized with PBS (1:9 ratio), then centrifuged at 5000×g for 10 minutes, and the supernatant was collected. Acetylcholine levels were detected using an AChE ELISA kit (Shanghai Scientific Biotechnology Co., Ltd.). After equilibrating the samples at room temperature for 60 minutes, standard and sample solutions were added to the wells, and the assay was conducted according to the kit instructions.

### 2.9 Aβ1–42 content measurement

Aβ1–42 levels were measured using pooled head tissues from 50 larvae per sample. Each experimental group was analyzed in triplicate biological replicates, washed with PBS, and weighed. The tissues were ground with PBS at a 1:9 ratio on ice. After centrifugation, the supernatant was collected for Aβ1–42 detection using the zebrafish Aβ1–42 ELISA kit (Shanghai Scientific Biotechnology Co., Ltd.). Standard wells, blank wells, and sample wells were set up, with different concentrations of standard solutions, samples, and HRP-labeled antibodies added. The wells were incubated at 37 °C for 60 minutes. After washing, substrate A and B were added, and the reaction was incubated at 37°C in the dark for 15 minutes. Finally, stop solution was added, and the OD values were measured at 450 nm.

### 2.10 Statistical analysis

A t-test and one-way ANOVA with Dunnett’s multiple comparison tests were applied to calculate the significance in qPCR data by the GraphPad Prism 9 software (GraphPad Software, San Diego California, USA). Data are shown as the mean ± standard deviation (SD), and p values < 0.05 were considered statistically significant.

## 3 Results

### 3.1 OMVs of *P. gingivalis* cause brain injury to zebrafish larvae

**Figure 1:**
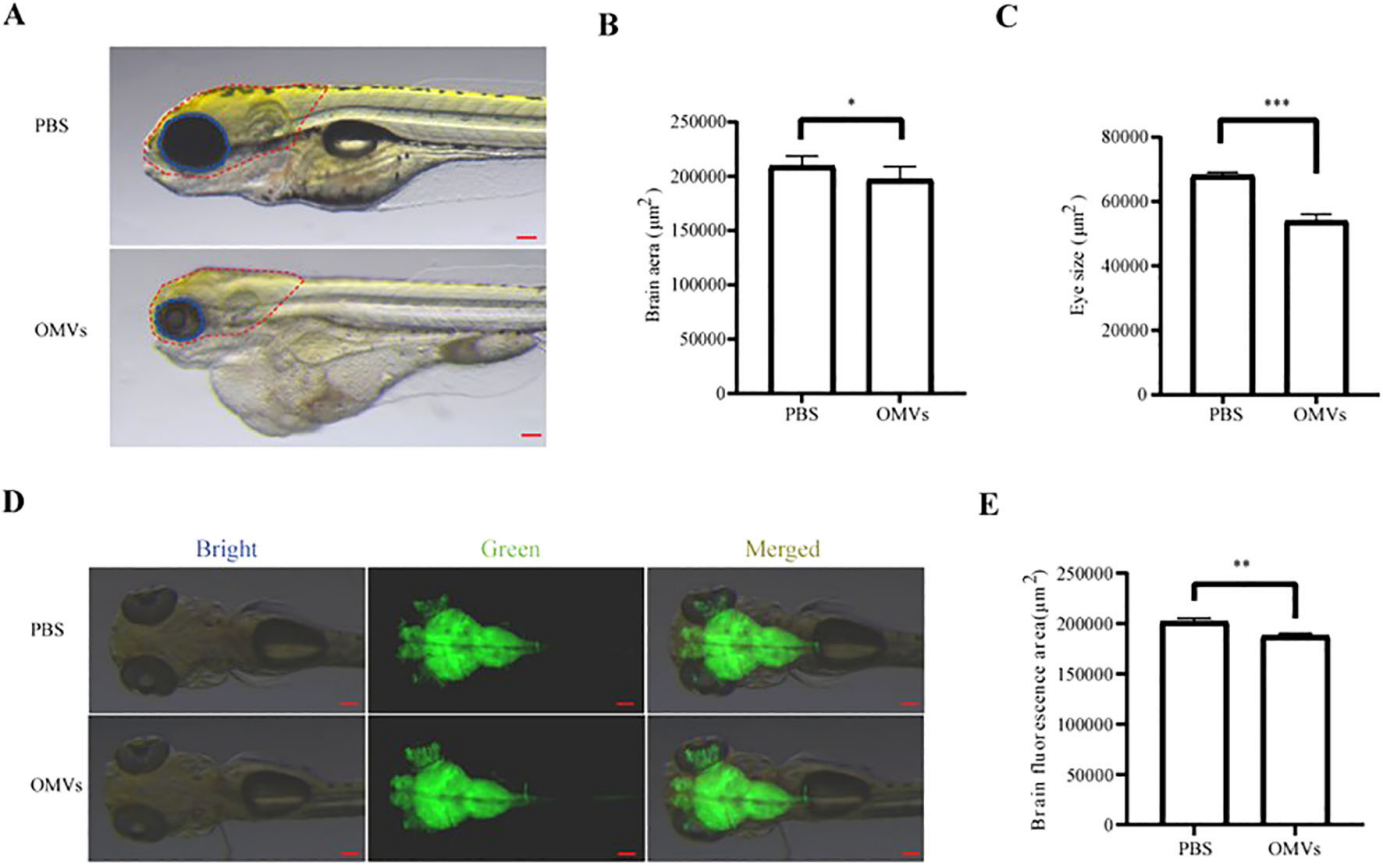
Analysis of the neurological impact of *Porphyromonas gingivalis* outer membrane vesicles (OMVs). **(A)** Representative diagram of head phenotype of zebrafish larvae at 48 hpi. Scale bar: 0.5 mm. **(B)** Brain area statistics for different treatment groups (PBS and 2.5 μg/μL) at 48 hpi. **(C)** Eye area statistics for different treatment groups (PBS and 2.5 μg/μL) at 48 hpi. **(D)** Image of vascular fluorescence at 2 hpi after treated with PBS or 2.5 μg/μL *P. gingivalis* OMVs. Live imaging was performed under a stereoscopic fluorescence microscope (SMZ800N) in the green channel. Scale bar: 0.5 mm. **(E)** Analysis results of neuronal fluorescence according to picture taken in **(D)** n=15, each group being replicated thrice. The unit is pixel, Mann Whitney test was used to analyze the significant difference between groups. *: *P* < 0.05; **: *P* < 0.01; ***: *P* < 0.001.

*P. gingivalis* OMVs were microinjected into the circulatory system of 48 hpf zebrafish larvae. As shown in **Supplementary Figure 1**, labeled OMVs were detected within the brain. After 48 hours, larvae were photographed using a stereomicroscope. The results demonstrated that the head area and eye size in the *P. gingivalis* OMVs-injected group were significantly smaller than those in the PBS-injected control group (*P*<0.05) (**Figures 1A-C**). To further verify the aforementioned phenomenon, the transgenic line expressing green fluorescent protein in neurons *Tg (elavl3:EGFP)* was utilized. Following the same procedure and analysis, the results revealed a reduced fluorescent area in the brain region of zebrafish larvae injected with *P. gingivalis* OMVs compared to the control group (*P*<0.01) (**Figures 1D, E**).

### 3.2 OMVs of *P. gingivalis* triggers inflammatory reactions to zebrafish brain

**Figure 2:**
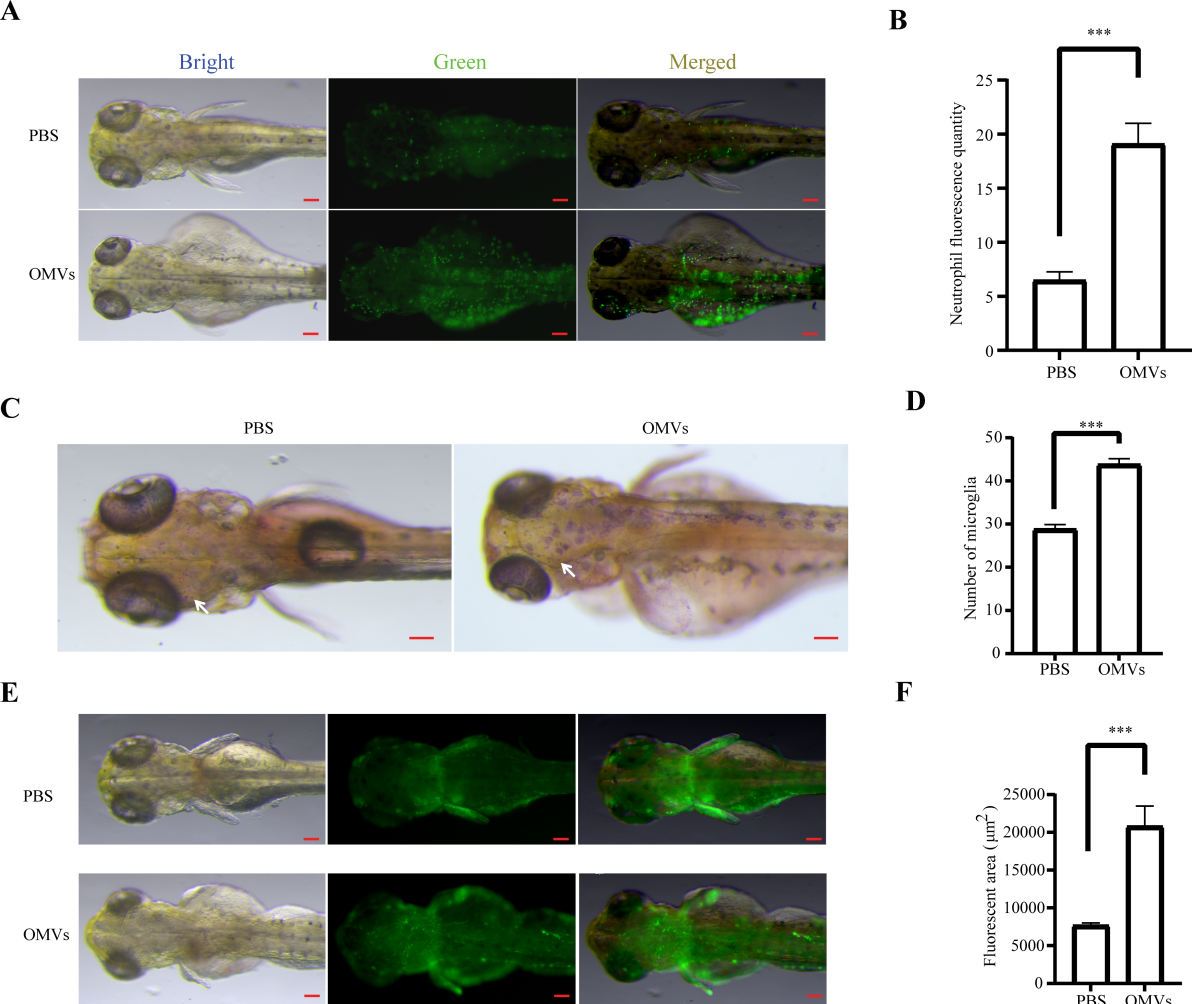
Analysis of the effects of *P. gingivalis* OMVs on neuroinflammation. **(A)** Image of neutrophils fluorescence at 48 hpi after treated with PBS or 2.5 μg/μL *P. gingivalis* OMVs. Scale bar: 0.5 mm. **(B)** Analysis results of neutrophils fluorescence according to picture taken in **(A)** n=15, each group being replicated thrice. Mann Whitney test was used to analyze the significant difference between groups, *P* < 0.001***. **(C)** Image of microglia recruitment at 48 hpi after treated with PBS or 2.5 μg/μL *P. gingivalis* OMVs. Scale bar: 0.5 mm. **(D)** Analysis results of neutrophils fluorescence according to picture taken in **(C)** n=15, each group being replicated thrice. Mann Whitney test was used to analyze the significant difference between groups, *P* < 0.001***. **(E)** Fluorescence image of NF-κB signaling pathway at 48 hpi after treated with PBS or 2.5 μg/μL *P. gingivalis* OMVs. Scale bar: 0.5 mm. **(F)** Analysis results of neutrophils fluorescence according to picture taken in **(E)** n=15, each group being replicated thrice. Mann Whitney test was used to analyze the significant difference between groups, *P* < 0.001***.

While bacterial-induced damage often initiates via inflammatory responses, the ability of *P. gingivalis* OMVs to cross the BBB and elicit neuroinflammation remains to be fully elucidated. To investigate this, we administered *P. gingivalis* OMVs to transgenic zebrafish reporter lines monitoring neutrophil infiltration *Tg(mpx:EGFP)* and NF-κB pathway activation *Tg(nfkb:EGFP)*. Our results demonstrate that *P. gingivalis* OMVs induce a significant recruitment of neutrophils and microglia within brain regions, concomitant with robust activation of the NF-κB signaling pathway (*P*<0.01) (**Figures 2A-F**). These findings indicate that *P. gingivalis* OMVs are capable of triggering a potent inflammatory response in the brain.

### 3.3 OMVs of *P. gingivalis* cause locomotor deficiency to zebrafish larvae

**Figure 3:**
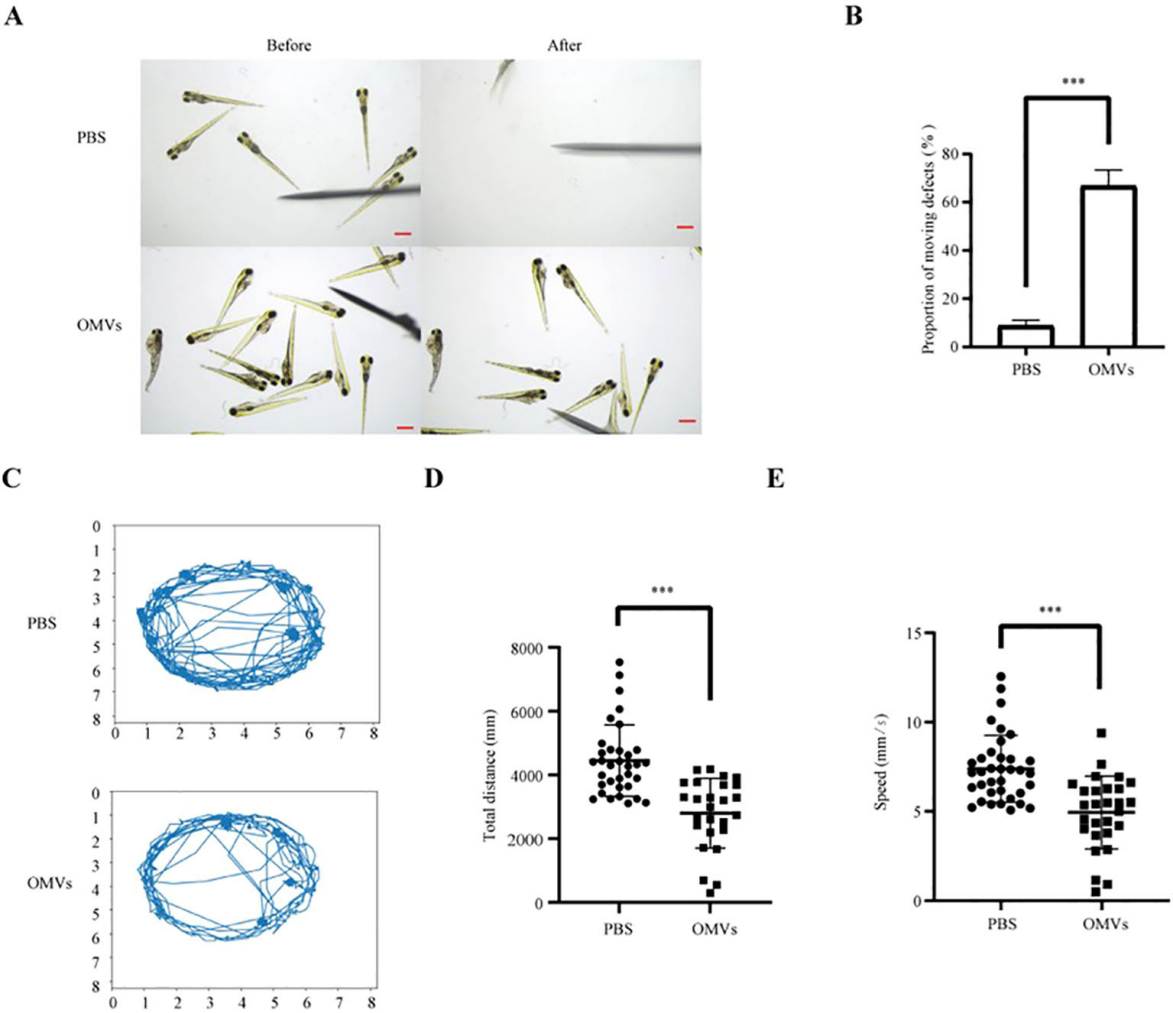
Analysis of the effect of *P. gingivalis* OMVs on the motility of zebrafish larvae. **(A)** Medication with PBS or 2.5 μg/μL *P. gingivalis* OMVs. Movement diagram of zebrafish touch-evoked escape response at 48 hpf after treatment with *P. gingivalis* OMVs. Scale: 0.5 mm. **(B)** Analysis of zebrafish Touch-evoked escape response based on photographs taken in Figure **(A)** n=15, three repetitions per group. Mann Whitney test was used to analyze the significant difference between groups, *P* < 0.01^**^ **(C)** Pair with PBS or 2.5 μg/μL *P. gingivalis* OMVs Movement trajectory analysis of zebrafish at 48 hpf after *P.gingivalis* OMVs treatment. **(D)** Analyze the total distance traveled based on the results of the analysis of the trajectory analysis diagram in **(C)** n = 15, three repetitions per group. The Mann Whitney test was used to analyze the significant differences between groups, *P* < 0.01^**^. **(E)** Analysis of movement speed based on the results of the analysis of the trajectory analysis diagram in **(C)** n = 15, three repetitions per group. The Mann Whitney test was used to analyze the significant differences between groups, *P* < 0.01^**^.

Motor function serves as a critical indicator of neural impairment. Tail-touch assays demonstrated that zebrafish larvae treated with *P. gingivalis* OMVs exhibited significantly reduced responsiveness to mechanical stimulation compared to the PBS control group (*P*<0.01) (**Figures 3A, B**). Furthermore, motion trajectory analysis revealed decreased total swimming distance and reduced average velocity in the OMV-treated cohort (*P*<0.01) (**Figures 3C-E**). These results collectively indicate that *P. gingivalis* OMVs induce motor deficits consistent with neural injury.

### 3.4 Proteomic and transcriptomic changes in P. gingivalis OMVs treated zebrafish

**Figure 4:**
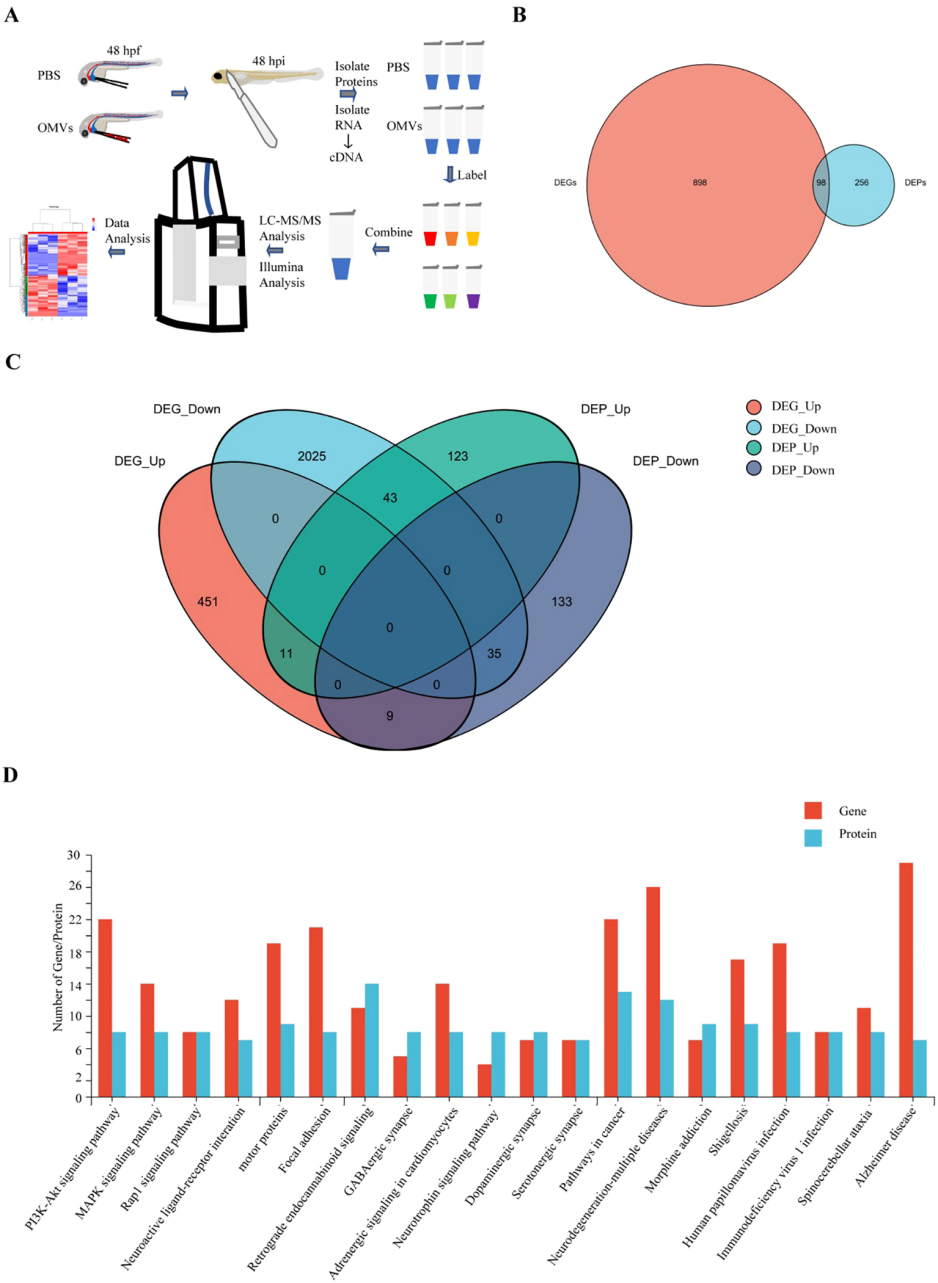
Proteomic and transcriptomic analysis. **(A)** Flowchart for joint analysis of transcriptomics and proteomics. **(B)** A total of 996 DEGs (FC > 2 or FC < 0.5) and 354 DEPs (FC > 1.2 or FC < 0.83) were identified between the OMVs injection group and the control group. **(C)** Venn diagram analysis revealed that 46 factors exhibited consistent expression trends at both the gene and protein levels, including 11 upregulated and 35 downregulated factors. **(D)** Comprehensive analysis of transcriptomics and proteomics with KEGG enrichment revealed the top 20 pathways with the most significant enrichment of DEGs and DEPs.

In this study, we aimed to explore the regulatory mechanisms of OMVs on AD-like changes in zebrafish by identifying differentially expressed genes (DEGs) and proteins (DEPs) through joint analysis of transcriptomics and proteomics (**Figure 4A**; **Supplementary Tables 1**-**5**). A total of 996 DEGs (FC > 2 or FC < 0.5) and 354 DEPs (FC > 1.2 or FC < 0.83) were identified between the OMVs injection group and the control group. Among these, 98 factors showed significant regulation at both the gene and protein levels (**Figure 4B**). Among the 98 regulated factors, 20 genes were upregulated and 78 genes were downregulated at the transcriptional level. At the protein level, 54 proteins were upregulated and 44 proteins were downregulated. Venn diagram analysis revealed that 46 factors exhibited consistent expression trends at both the gene and protein levels, including 11 upregulated and 35 downregulated factors (**Figure 4C**). Integrated Multi-Omics Analysis Reveals the Core Pathway Mechanisms of AD - Like Lesions Induced by *P. gingivalis* in Zebrafish Through the integrated analysis of transcriptomics and proteomics with KEGG enrichment, we identified the top 20 most significantly enriched pathways with differentially expressed genes (DEGs) and proteins (DEPs) in zebrafish at 48 hpf following treatment with *P. gingivalis*. As shown in the figure, the Alzheimer’s disease pathway and neurodegenerative diseases exhibited synergistic enrichment at both the gene and protein levels (**Figure 4D**). Additionally, the PI3K-Akt signaling pathway and the MAPK signaling pathway were significantly dysregulated. The dual enrichment of the Alzheimer’s disease pathway is directly associated with the core neuropathological features: β-amyloid deposition, hyperphosphorylation of Tau protein, and synaptic dysfunction. Specifically, the dysregulation of the PI3K-Akt pathway leads to impaired neuronal trophic support and increased susceptibility to apoptosis (Liu et al., 2021; Zhou et al., 2021). Furthermore, the MAPKs-NF-κB signaling pathway induces microglia-mediated neuroinflammation, which amplifies neuroinflammatory toxicity (Liu et al., 2020). The mass spectrometry proteomics data have been deposited to the ProteomeXchange Consortium (https://proteomecentral.proteomexchange.org), with the dataset identifier PXD067862. The transcriptomics data can be accessed via https://www.ncbi.nlm.nih.gov/sra/PRJNA1311755, corresponding to the project PRJNA1311755.

**Figure 5:**
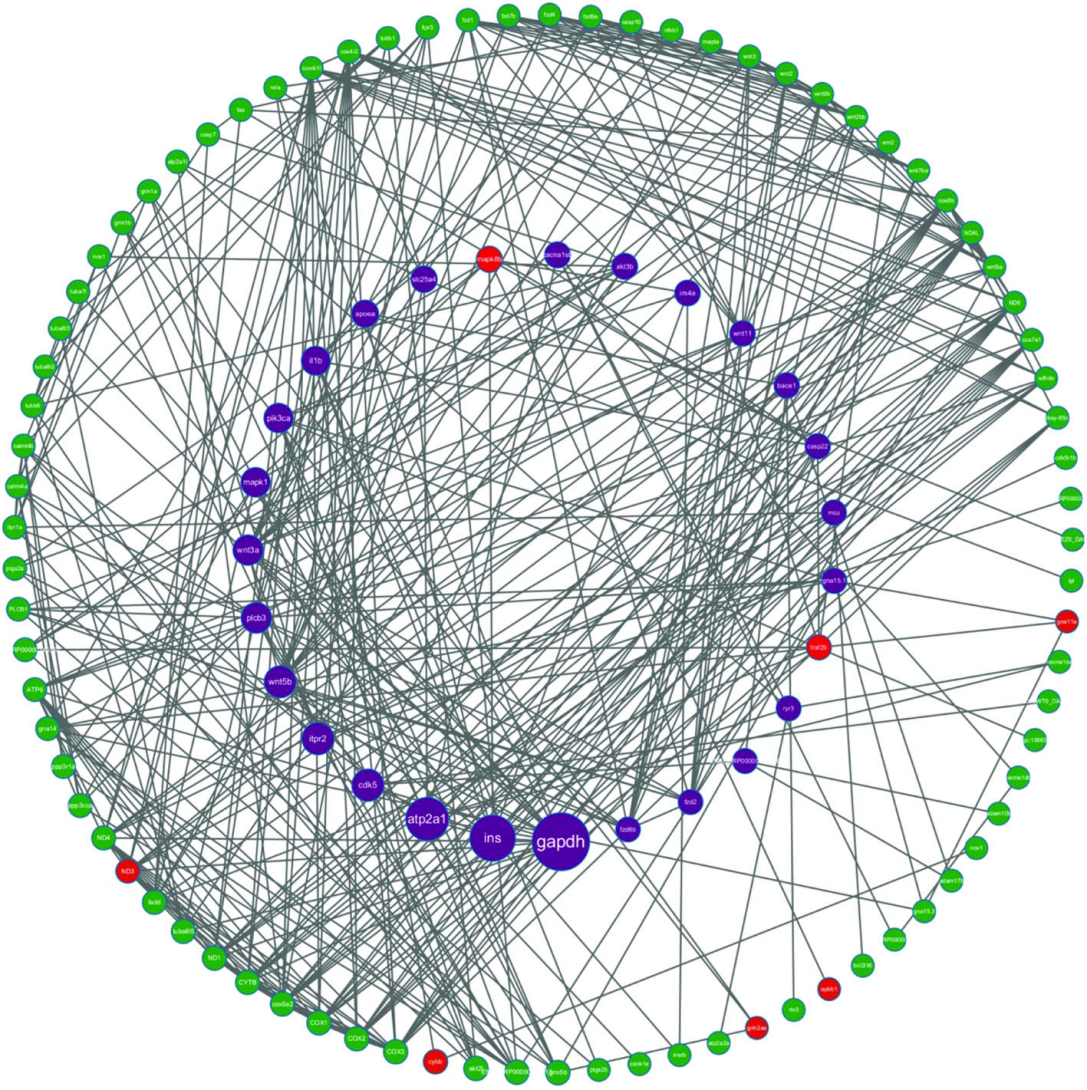
The protein-protein interaction network. Nodes represent genes, and edges indicate the interactions between proteins encoded by these genes. Node colors denote gene categories: green for examined genes, purple for key genes (e.g., ins, gapdh), and red for genes with significant roles in the network.

Identification and Analysis of Key Genes in the AD Pathway in Zebrafish Using RNA-seq technology, we identified 128 DEGs annotated to the AD pathway (KEGG annotation). Based on these genes, a protein-protein interaction (PPI) network was constructed using the STRING database, with a medium confidence threshold set at an interaction score ≥0.4 (**Figure 5**). The final PPI network consisted of 125 nodes and 382 edges. Based on these findings, combined with the proteomic analysis, we further identified multiple key differentially expressed proteins closely associated with the Alzheimer’s disease (AD) pathway, including apbb1, mapk8b, traf2, gna11a, grin2aa, cybb, and mt-nd3(**Supplementary Figure 2**). Notably, traf2 was present both in the AD pathway-enriched DEG set and exhibited relatively high betweenness centrality within the PPI network, suggesting a potentially central role in network information flow and signal integration compared to other candidate targets.

### 3.5 Effects of P. gingivalis OMVs on AChE activity and Aβ1–42 plaques

**Figure 6:**
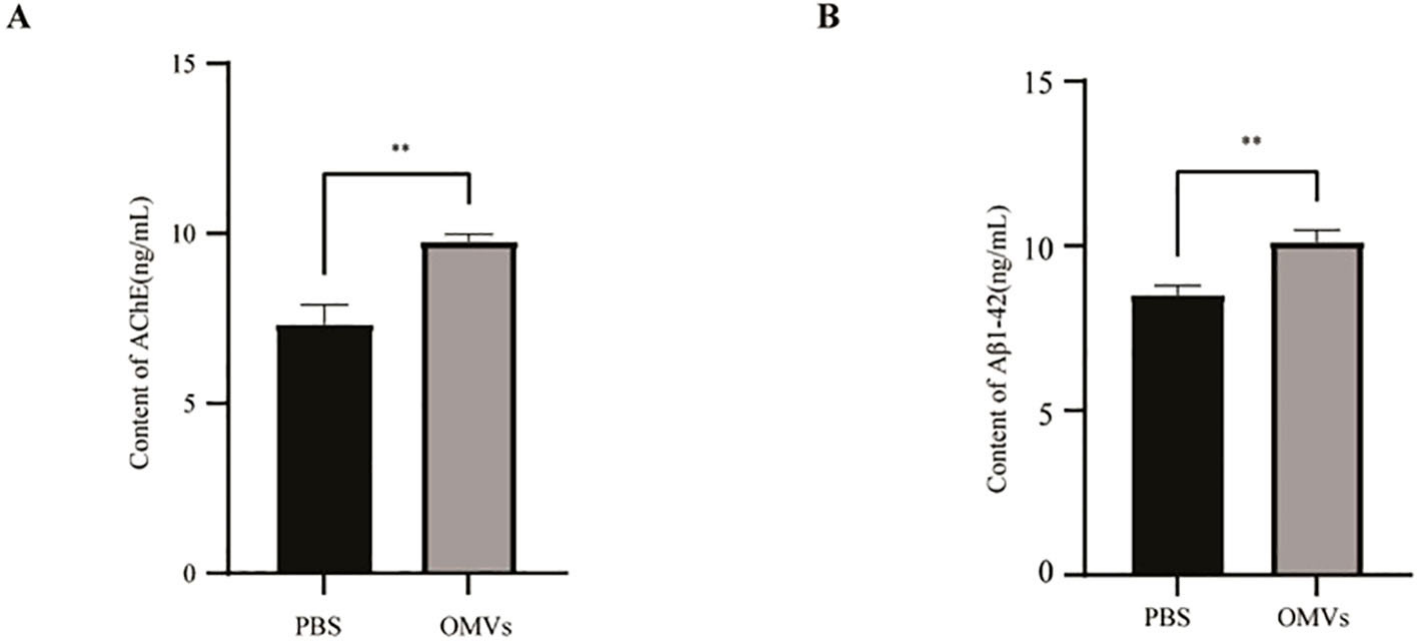
Determination of AChE activity and Aβ1–42 content. A, B: Quantitative analysis of AChE fluorescence intensity **(A)** and Aβ1–42 plaques **(B)** 2 dpi zebrafish to PBS and OMVs. Data are presented as the mean ± SEM (n = 3). Statistical significance of treatment effects was assessed using one-way ANOVA with Dunnett’s multiple comparison test and illustrated as follows: *P* < 0.01^**^.

AChE, an important enzyme responsible for acetylcholine hydrolysis in the brain and inducing cholinergic neuronal dysfunction, is currently the primary target of AD therapy (Uddin et al., 2021). Therefore, after treatment with *P. gingivalis* OMV, we determined the AChE activity of 48 hpi zebrafish to confirm successful construction of an AD-related cholinergic impairment. We found that AChE level, as determined by ELISA,was significantly increased in zebrafish larvae treated with *P. gingivalis* OMVs compared with the normal control group. (**Figure 6A**). This finding aligns with the observed behavioral performance of zebrafish as discussed above. Aβ deposition is an important clinical hallmark in patients with AD. We next examined the level of Aβ1–42, a highly neurotoxic amyloid peptide that accumulates in the brains of patients with AD (Dou et al., 2024). Only few Aβ1–42 plaques were observed in the control group (**Figure 6A**). In contrast, there was a notable increase in the content of Aβ1–42 plaques observed in zebrafish treated with *P. gingivalis* OMVs (**Figure 6B**). This increasing trend was consistent with the increase in AChE activity, suggesting that exposure to *P. gingivalis* OMVs may induce AD-like manifestations in zebrafish.

## 4 Discussion

Periodontitis is a chronic infectious disease associated with plaque biofilms, leading to the progressive destruction of periodontal tissues. AD, a neurodegenerative disorder with complex multifactorial pathology, has been linked to chronic periodontitis. Emerging evidence suggests that periodontitis may increase AD risk, with *P. gingivalis*, a key pathogen in periodontal dysbiosis, playing a central role (Liu et al., 2018; Marruganti et al., 2023). This bacterium secretes OMVs, that, due to their nanoscale size, strong adhesiveness, stability, and virulence factor enrichment, may mediate distant pathological effects. In this study, we used a zebrafish model to show that *P. gingivalis*-derived OMVs directly impair brain structures, induce neuroinflammation, and lead to AD-like pathology, including Aβ plaque deposition and increased AChE activity. These findings provide critical insight into the pathogenic mechanisms linking periodontitis to AD, which may guide the development of targeted therapeutic strategies.

In this study, we first investigated the effects of *P. gingivalis* OMVs on zebrafish brain development by microinjecting varying concentrations of OMVs into the common cardinal vein of zebrafish embryos. Compared with controls, treated embryos exhibited significant reductions in brain and ocular volume. Behavioral assays revealed that OMV-exposed larvae displayed decreased total locomotor distance and reduced swimming speed. These results indicate that *P. gingivalis* OMVs may induce neural damage via direct invasion or disruption of the BBB. Produced during the growth of P. gingivalis, OMVs carry a diverse array of virulence-associated components, including LPS, pili, peptidylarginine deiminase (PPAD), gingipains, and regulatory small RNAs (sRNAs). Although not all surface factors are uniformly enriched, OMVs selectively concentrate several potent virulence proteins—particularly gingipains and other outer-membrane–associated enzymes—thereby conferring a higher per-particle virulence potential and enabling OMVs to penetrate deeper tissues more effectively than the parent bacteria. Their small size and stability enable deeper tissue penetration and robust activation of host inflammatory responses (O’Brien-Simpson et al., 2009). Consequently, OMVs can disseminate to distal host sites ordinarily inaccessible to bacteria, amplifying pathogenic effects both locally and systemically (Farrugia et al., 2020). This dissemination provides a mechanistic explanation for how oral microbes induce neurotoxicity at distant sites, specifically in the brain. We attribute the observed neurophenotypes in OMV-injected zebrafish to the direct neurotoxic effects of these vesicles. Previous *in vitro* studies have demonstrated that OMV-associated gingipains can damage neuronal structures via clathrin-mediated endocytosis (Chuang et al., 2024).

Previous studies have demonstrated that *P. gingivalis* OMVs induce neuroinflammation. After endocytosis by microglia, OMV-associated LPS activates the AKT/JNK signaling pathway, markedly enhancing the secretion of proinflammatory cytokines such as IL-6 and TNF-α (Nonaka et al., 2022). Concurrently, OMVs trigger NLRP3 inflammasome assembly, resulting in excessive cytokine release within the central nervous system and a feed-forward amplification of neuroinflammatory responses that establishes a persistent proinflammatory milieu. Additionally, gingipains enriched within *P. gingivalis* OMVs exhibit notable neurotoxicity; these enzymes specifically degrade critical extracellular matrix components of neurons, such as laminin and fibronectin, thereby disrupting neuronal support structures. This degradation not only impairs the functional connectivity between neurons and glial cells but also directly induces neuronal apoptosis, further exacerbating the vicious cycle of neuroinflammation (Dominy et al., 2019). Our zebrafish model provides direct *in vivo* evidence for this mechanism: *P. gingivalis* OMVs exposure significantly increases neutrophil and microglial counts compared with controls, indicating microglial activation within the brain. Proteomic analysis corroborates these findings, revealing upregulation of inflammation-related proteins such as mapk8b and cybb. These results align with prior work by Jiang et al., who showed that OMVs induce microglial activation and elevate IL-1β, TNF-α, IL-6, and iNOS in a wild-type mouse model (Jiang et al., 2025). Furthermore, our previous research established that *P. gingivalis* OMVs directly induce cardiac injury—manifesting as pericardial enlargement, vascular damage, and inflammatory responses—thereby underscoring a common mechanism by which OMVs mediate distal tissue damage and inflammation (Guo et al., 2024).

The pathogenesis of AD is multifactorial, encompassing hypotheses such as the cholinergic hypothesis (Liang et al., 2022), the amyloid cascade hypothesis (Lopez Sanchez et al., 2019), tau hyperphosphorylation (Dong et al., 2022), oxidative stress (Cheignon et al., 2018), neuroinflammation (Liu et al., 2018), and blood-brain barrier disruption (Kazemeini et al., 2024). Among these, cholinergic neuron degeneration is a pivotal contributor to cognitive decline. Acetylcholine (AChE), the primary cholinergic neurotransmitter, plays a vital role in regulating physiological processes such as attention, learning, memory, stress response, the sleep-wake cycle, and sensory information processing (Hasselmo et al., 1992; Fine et al., 1997; Sarter and Bruno, 1997; Miranda and Bermúdez-Rattoni, 1999; Haam and Yakel, 2017). The amyloid cascade hypothesis further posits that aberrant Aβ accumulation initiates AD pathology by promoting extracellular plaque formation, intracellular neurofibrillary tangles, and widespread neuronal loss (Hardy and Higgins, 1992; De Strooper and Karran, 2016); accordingly, Aβ remains a key therapeutic target. To assess whether *P. gingivalis* OMVs elicit AD−like features, we quantified AChE activity and Aβ deposition in zebrafish brains. OMV−treated larvae exhibited significant elevations in both markers, indicating induction of AD−relevant pathology. Complementary KEGG pathway annotation analysis revealed enrichment of neurodegenerative disease-related pathways following OMV exposure. Collectively, these data provide molecular evidence that *P. gingivalis* OMVs modulate cholinergic dysfunction and Aβ pathology *in vivo*.

In AD research, the zebrafish model offers several advantages. The zebrafish genome shares over 80% homology with human disease-associated genes, and cross-species single-cell and nuclear transcriptomic analyses by Cosacak et al. demonstrated 95.4% conservation of neuronal molecular responses in an Aβ−42–induced zebrafish AD-related cholinergic impairment (Howe et al., 2013; Cosacak et al., 2022). The optical transparency of zebrafish larvae permits real−time, noninvasive imaging of craniofacial and neural structures, and genetic and pharmacological manipulations are readily achievable. Moreover, zebrafish enable high−throughput screening at low cost (Kimmel et al., 2001; Hruscha et al., 2013). Accordingly, we employed zebrafish *in vivo* to investigate OMV−induced neurodegeneration. Building on our prior work demonstrating cardiovascular toxicity of *P. gingivalis* OMVs (Guo et al., 2024), we extended this model to neurodegenerative disease, establishing its capacity to recapitulate AD−like pathology.

Despite rigorous experimental design, this study has several limitations. First, anatomical and developmental differences in brain structure and BBB formation between zebrafish and mammals may affect translatability. Second, as the zebrafish BBB is not fully mature during a transitional stage between initial barrier formation (2–3 dpf) and full maturation (10 dpf) (Xie et al., 2010; Fleming et al., 2013), its relative immaturity may influence permeability characteristics and should be considered when interpreting OMV-associated effects. Future studies should therefore include fully mature stages and a range of OMV doses, as well as validate the findings in mammalian models to enhance rigor and translational relevance, thereby providing deeper insights into the mechanisms underlying the effects of OMVs. Third, the present study was conducted within a 48-hour post-injection window and future studies employing chronic OMV exposure models should be necessary to elucidate the temporal evolution from acute molecular disturbances to progressive neurodegenerative pathology. Moreover, intravenous microinjection bypasses physiological BBB permeation, potentially introducing systemic bias by failing to model gradual Aβ accumulation through endogenous transport mechanisms. Finally, particle-driven effects independent of OMV cargo cannot be completely ruled out. Future studies should incorporate inert particulate controls or inactivated OMVs to better distinguish OMV-specific signaling from nonspecific responses related to particulate internalization (Uemura et al., 2022; Zhang et al., 2022).

To clarify the mechanistic link between *P. gingivalis* OMVs and AD, future studies should address the following priorities. First, OMVs have previously been shown to induce cardiovascular injury in this zebrafish model (Guo et al., 2024); which could secondarily affect neural development through altered perfusion or systemic inflammation. The identification of OMV-induced central nervous system alterations in this study was supported by specific molecular indicators associated with AD-like pathology, including changes in AChE activity and Aβ1–42 levels. In addition, the detection of OMVs within brain tissue further supports a direct interaction between OMVs and the central nervous system. Future studies designed to quantify inflammatory cytokines in the brain and peripheral tissues could help elucidate the neuroinflammatory responses triggered by OMVs. Second, identify and characterize specific OMV components that drive AD pathology. Third, develop and screen targeted inhibitors that block OMV−mediated neurotoxicity as candidate therapeutics. Additionally, integrate multi−omics datasets to construct a comprehensive molecular regulatory network. This study offers pioneering *in vivo* evidence in zebrafish that OMVs mediate the periodontal disease–AD axis, establishing a foundation for an “oral–brain axis” intervention framework. By elucidating how periodontitis—and specifically *P. gingivalis* OMVs—impacts neural systems, future work may reveal novel biomarkers for early AD diagnosis, inform precision treatment strategies, and guide preventive measures through targeted modulation of OMV pathways.

## 5 Conclusions

Our study demonstrates that *P. gingivalis* OMVs induce neurotoxicity and behavioral impairments in zebrafish larvae. Exposure to OMVs caused significant brain damage, neuroinflammation, and locomotor dysfunction. Using proteomic and transcriptomic approaches, we identified molecular changes linked to AD, suggesting that OMVs activate AD-related disease mechanisms. Experimental validation showed increased AChE activity and Aβ1–42 plaque accumulation, key features of AD pathology. These findings establish *P. gingivalis* OMVs as potent inducers of neuronal injury, neuroinflammation, and AD-like changes, supporting a mechanistic link between periodontal pathogens and neurodegenerative processes. This highlights the importance of oral health in systemic and neurological disease.

## Data Availability

The datasets presented in this study can be found in online repositories. The names of the repository/repositories and accession number(s) can be found in the article/**Supplementary Material**.
