# Supplementary material for: Zebrafish study provides evidence for Porphyromonas gingivalis outer membrane vesicles eliciting Alzheimer’s disease-like pathologies
Source: Front Cell Infect Microbiol. 2026 Mar 6;16:1761068. doi: 10.3389/fcimb.2026.1761068 (PMC13002584; doi:10.3389/fcimb.2026.1761068)
Supplement: Supplementary file 1 [file Table1.docx]

Supplementary Material


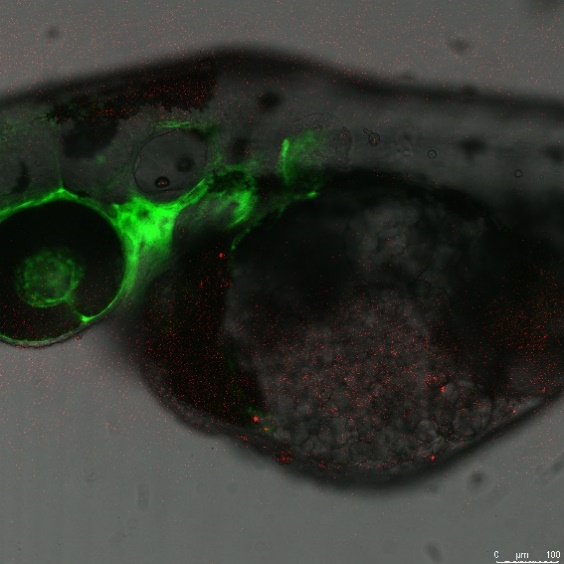


Figure S1. Localization of P. gingivalis OMVs in brain tissue. Representative fluorescence image showing the distribution of labeled P. gingivalis OMVs. Red fluorescence indicates P. gingivalis OMVs. Scale bar: 100 μm.


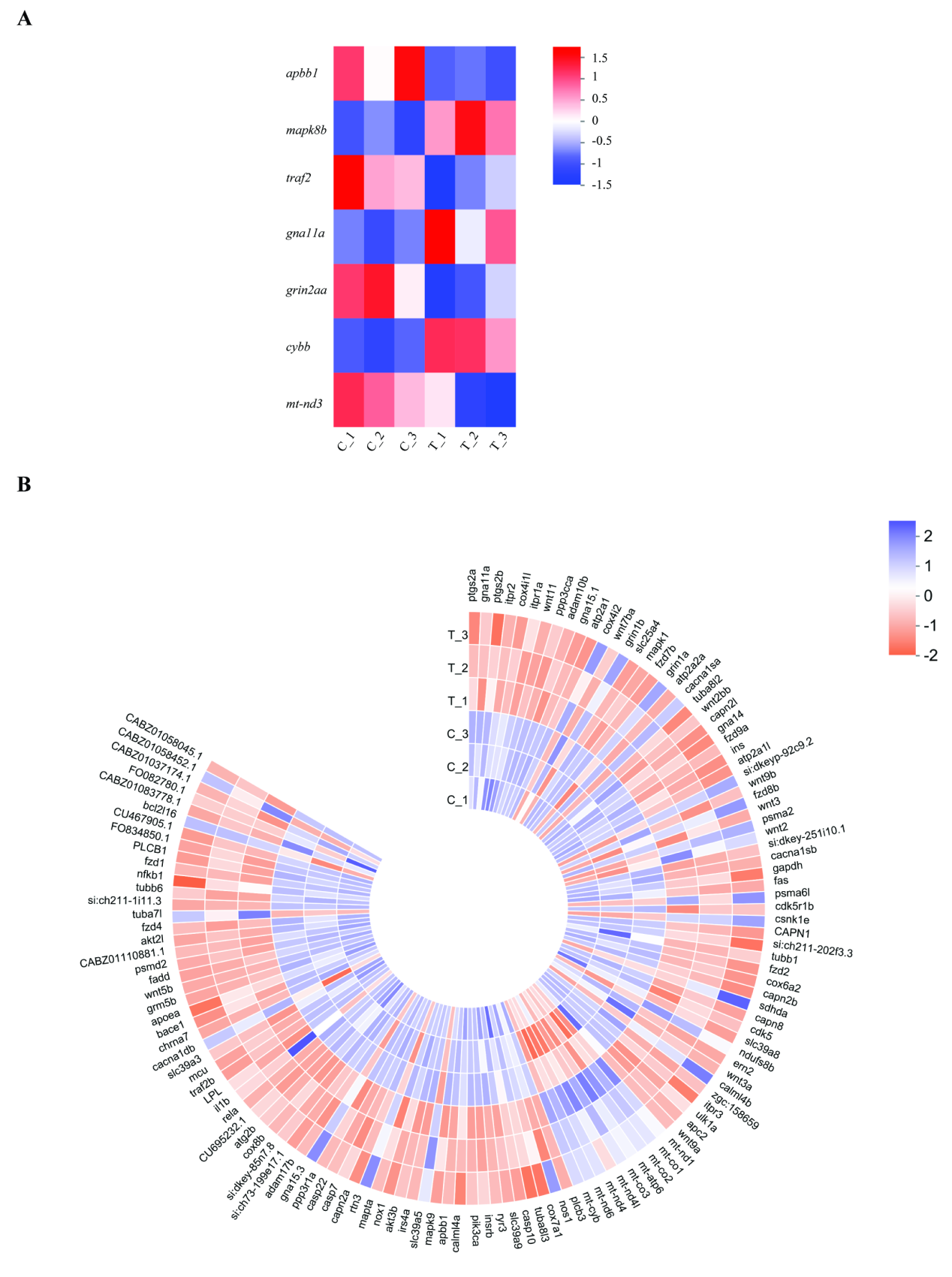


Fig. S2.

**Supplementary table1：DEGs+DEPs**

S**upplementary table2：DEGs (Up)+DEPs (Up)**

**Supplementary table3：DEGs (Up)+DEPs (Down)**

**Supplementary table4：DEGs (Down)+DEPs (Up)**

**Supplementary table5：DEGs (Down)+DEPs (Down)**
